# Influence of an Alternative Diagnosis on the Diagnosis of Pulmonary Thromboembolism

**DOI:** 10.3390/healthcare12222246

**Published:** 2024-11-11

**Authors:** Rafael Suárez del Villar Carrero, Diego Martínez-Urbistondo, Miguel De la Serna Real de Asúa, Ángel Cano Mazarro, María Agud Fernández, Ana Rodríguez Cobo, Paula Villares Fernández

**Affiliations:** 1 Department of Internal Medicine Hospital HM Sanchinarro, HM Hospitales, Avenida/Montepríncipe 26, 28260 Madrid, Spain; 2 Internal Medicine Department, University Clinic of Navarra, 28027 Madrid, Spain; 3 Faculty of Medicine, University San Pablo CEU, 28925 Madrid, Spain

**Keywords:** pulmonary thromboembolism, clinical decision rule, Wells scale, Pulmonary Embolism Rule-Out Criteria

## Abstract

Background: The diagnosis of pulmonary embolism (PE) is based on the application of a priori probability scales such as the Wells scale or PERC. However, the clinical heterogeneity of this pathology results in the absence of a target population to apply these algorithms. The Wells score does consider the possibility of an alternative diagnosis, awarding an additional point if no other diagnosis is likely, yet the presence of objective alternative diagnoses can still complicate clinical assessment and lead to unnecessary testing or missed diagnoses. Objective: The aim of this study is to evaluate the discrimination capacity of clinical objective factors with a high negative predictive value for PE, compared to PERC in terms of reducing unnecessary testing across different risk strata of the Wells scale. Materials and Methods: This was a single-center retrospective cohort study, including patients who underwent chest CT angiography to rule out PE at a university hospital between 2008 and 2017, considering the presence of PE as the study outcome. The study collected demographic data, comorbidities, and clinical presentation data. The presence of objective criteria for pneumonia, heart failure, exacerbation of COPD, or the use of anticoagulation in non-oncological patients were considered a priori criteria with a high negative predictive value. Results: The analyses were performed on a cohort of 399 patients with an average age of 65 years and 53% females. A total of 139 patients were diagnosed with PE by CT angiography. The presence of factors with a high NPV showed a sensitivity of 100% in low-risk patients according to Wells, with sensitivity dropping below 50% in other populations. The association of these factors in the PERC plus criteria would allow a reduction of up to 34% in CT angiographies in patients with low risk according to the Wells scale. Conclusions: The combination of risk stratification of the Wells scale and PERC plus criteria allows an absolute reduction of 34.3% in the performance of CT angiographies in patients classified as low risk with a sensitivity and a negative predictive value of 100%. The preexistence of an alternative diagnosis does not allow ruling out PE in patients with intermediate or high risk according to the Wells scale.

## 1. Introduction

Pulmonary embolism (PE) is a prevalent condition, with an annual incidence ranging from 39 to 115 cases per 100,000 inhabitants [1,2,3,4]. It is the third most common cardiovascular disease, following acute myocardial infarction and cerebrovascular accident [5,6,7], with morbidity and mortality rates reaching up to 30% in some reports [2].

The clinical presentation of PE is highly variable and non-specific [8,9,10,11]. Most commonly, it presents with dyspnea, cough, or chest pain [10,12], symptoms that overlap with other pulmonary and cardiovascular conditions such as pneumonia, exacerbation of COPD, or decompensated congestive heart failure, among others [9,11,13]. Additionally, the average age of PE presentation is between 60 and 70 years, a period when these other pathologies are also prevalent [1,13,14,15,16,17,18,19]. Consequently, establishing a presumptive diagnosis of PE based solely on clinical and epidemiological data can be challenging [8,20,21]. Several risk factors for venous thromboembolic disease (VTE) can modify the probability that a patient’s symptoms are due to PE. Notably, cancer, recent surgery, immobility, and estrogen use are associated with an increased risk of PE [22,23,24,25].

Clinical prediction scales have been developed to stratify the risk of PE in patients. Among these, the Wells score is widely used and has demonstrated strong external validation, serving as a primary tool for initial PE risk assessment [7,26]. This scale evaluates signs, symptoms, and risk factors, categorizing patients into low, medium, or high risk [7,26]. Based on this assessment, patients are directed through clinical algorithms to undergo further tests for diagnosis or exclusion of PE [21,27,28,29,30]. However, the Wells score has limitations, including its exclusion of hospitalized patients, lack of a clearly defined target population, inclusion of subjective elements, and dependence on a hospital setting capable of performing necessary tests and procedures [31].

More recently, alternative approaches to diagnosing VTE have emerged. The Pulmonary Embolism Rule-Out Criteria (PERC) make up one such model, including eight factors with a high negative predictive value for PE [32]. This model can rule out PE in patients with very low probability, achieving near 100% sensitivity [33,34,35]. Despite their usefulness, PERC’s representativeness is limited, affecting approximately 13% of the at-risk population and thereby being inadequate for many patients with suspected PE [36].

In summary, the epidemiological, clinical, and analytical heterogeneity of PE patients, combined with the complexity of diagnostic tests, complicates standardized clinical judgment. The presence of concurrent diagnoses, such as pneumonia, COPD exacerbation, or congestive heart failure, can further complicate clinical evaluations and challenge the effectiveness of existing prediction models like the Wells scale and PERC. While it seems reasonable to rule out PE in patients with a clear alternative diagnosis, such as pneumonia, COPD exacerbation, or congestive heart failure, or those on anticoagulant therapy for other reasons [37], definitive evidence supporting this approach is lacking. Therefore, this study aims to evaluate the discrimination capability of clinical factors with a high negative predictive value for PE and compare them to the PERC scale in terms of screening efficacy and reduction in unnecessary testing.

Concurrent diagnoses are not unique to PE, as other conditions with overlapping symptoms can complicate timely diagnosis and management. For instance, a large study on severe COVID-19 cases revealed a 30% rate of concurrent diagnoses, leading to delays and highlighting the importance of assessing multiple potential diagnoses in complex clinical scenarios [38]. This underscores the need for a thorough and structured approach to diagnostic evaluation in patients with suspected PE and potential alternative diagnoses.

## 2. Materials and Methods

**Figure 1:**
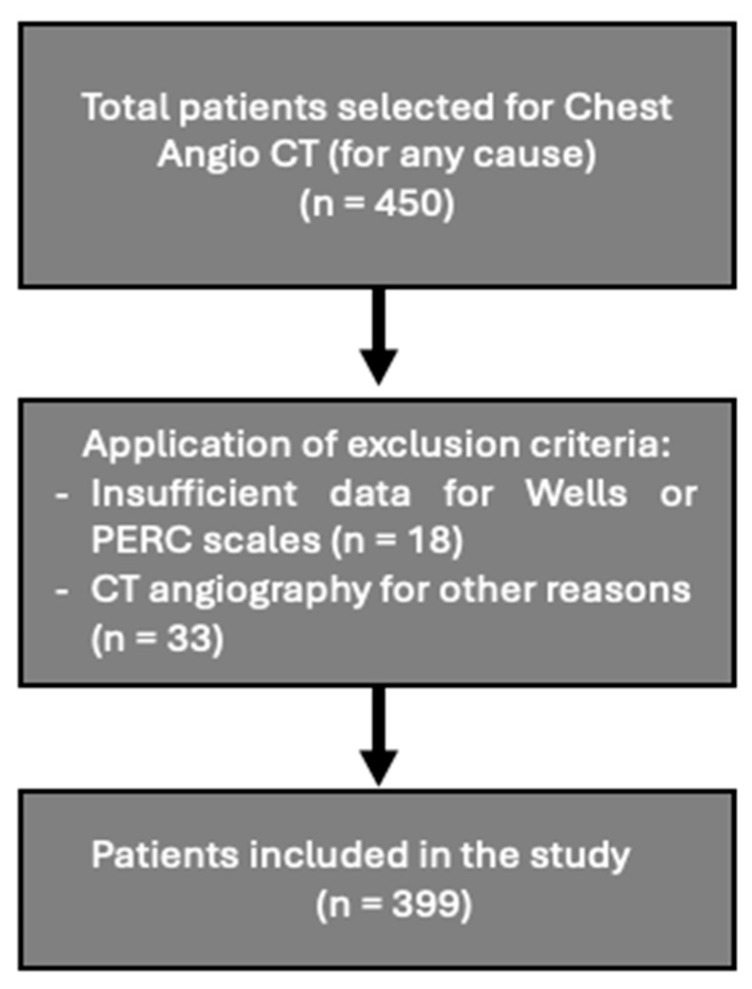
Flowchat of patient selection.

This observational, retrospective cohort study included all patients who underwent chest computed tomography angiography (CT angiography) between 2008 and 2017 at HM Sanchinarro Hospital (a 200-bed university hospital), were above 18 years of age, and had sufficient data for calculating the Wells or PERC scales. Exclusion criteria involved patients who had an acute PE diagnosis at the time of the test or underwent CT angiography for reasons other than suspected PE. A flowchart (Figure 1) depicting the patient selection process has been included for further clarity. The study was approved by the center’s ethics committee, and informed consent was waived (HM Hospitals CEIm Code: 21.03.1792-GHM). 

Patients were identified through a review of digitized medical records. Data collection included demographic information, risk factors, comorbidities, clinical variables, vital signs at the time of the test, and analytical and radiographic data. Variables corresponding to the Wells and PERC scales (Table 1 and Table 2) [26,32] were recorded. To be considered positive by the PERC, a patient must meet all eight criteria. These criteria are used to rule out PE in patients with very low clinical probability. The presence of PE on CT angiography was considered the outcome variable. This resulted in a final study cohort of 399 patients.

Objective variables with a high negative predictive value were defined based on diagnostic guidelines [39,40,41]: (i) Pneumonia: fever and radiographic consolidation as assessed by the physician; (ii) COPD exacerbation: smoking habit, cough, and fever; (iii) congestive heart failure (CHF): radiological pattern consistent with heart failure and dyspnea or orthopnea; (iv) previous anticoagulation in non-oncological patients: anticoagulated patients without a prior oncological diagnosis at the time of CT angiography (Table 3).

Statistical analysis employed standard methods. Qualitative variables were analyzed using the chi-square test, and quantitative variables were assessed with *t*-tests and ANOVA. Sensitivity, specificity, and positive and negative predictive values were calculated for each study variable and the PERC scale. A new scale, PERC plus, incorporating all variables with a 100% negative predictive value, was created. The ability of each criterion and the PERC plus set to reduce diagnostic tests was assessed by calculating the incidence of at-risk patients where CT angiography could have been avoided. Differences with *p* < 0.05 were considered statistically significant. Statistical analysis was performed using SPSS version 27.0 (Armonk, NY, USA, 2020).

## 3. Results

The study evaluated 399 patients who met the inclusion criteria, with 139 (35%) diagnosed with PE by CT angiography. The cohort had an average age of 65 ± 16 years, and 53% were female (*n* = 214). The average number of comorbidities per patient was 0.8 ± 0.3, with oncological pathology being the most common (*n* = 121, 30%). Risk stratification according to the Wells scale showed that 6 patients with PE (4%) were in the low-risk group, 95 patients (68%) were in the moderate-risk group, and 38 patients (27%) were in the high-risk group. CT angiography was performed after emergency department assessment in 81.20% of patients.

The most frequent symptoms were dyspnea (*n* = 269, 67%), pleuritic pain (*n* = 154, 39%), cough (*n* = 115, 29%), fever (*n* = 66, 17%), lower limb pain (*n* = 43, 11%), orthopnea (*n* = 25, 6%), syncope (*n* = 15, 4%), and hemoptysis (*n* = 11, 3%). At diagnosis, 108 patients (27%) had hemodynamic instability, and 185 patients (46%) had acute respiratory failure. PERC were met by 13 patients (3%), pneumonia criteria by 27 patients (7%), COPD exacerbation criteria by 40 patients (10%), CHF criteria by 18 patients (5%), and 29 patients (7%) were anticoagulated without oncological pathology. The distribution of these patients according to Wells scale risk is shown in Table 4.

Patients with PE (*n* = 139) had an average age of 66 ± 17 years and 47% were female (*n* = 65), while non-PE patients (*n* = 260) had an average age of 64 ± 16 years and 57% were female (*n* = 149), showing no statistically significant differences. The average number of comorbidities in PE patients was 0.9 ± 0.3, with a predominance of oncological pathology (*n* = 45, 32%), like the non-PE group. PE patients were more frequently detected in the emergency department (*n* = 124, 89.2%) and were associated with lower limb pain (22.30% vs. 4.60%, *p* < 0.001), heart rate > 100 bpm (33.10% vs. 22.70%, *p* = 0.025), and oxygen saturation < 94% (56.80% vs. 40.80%, *p* = 0.002). Cough was associated with negative CT angiography (15.10% vs. 36.20%, *p* < 0.001). Hemodynamic instability and desaturation were more frequent in PE patients. Both the PERC scale and the presence of COPD exacerbation and anticoagulation in non-oncological patients showed discrimination for PE (*p* < 0.05), while pneumonia criteria approached statistical significance (*p* = 0.06). CHF criteria did not show a significant association with PE (*p* = 0.71) (Table 5).

In Table 6, we present the sensitivity, specificity, positive predictive value (PPV), and negative predictive value (NPV) of each clinical criterion with a high a priori negative predictive value for PE. Sensitivity here refers to the percentage of patients correctly identified as having PE among those who indeed have the condition, while specificity indicates the percentage of correctly identified patients without PE among those who do not present with the condition. For instance, the 100% sensitivity observed for pneumonia in low-risk groups suggests that all patients in this category who did not have PE also met the pneumonia criteria, indicating that pneumonia may serve as an effective exclusion criterion within this subgroup.

The NPV and PPV calculations further illustrate the diagnostic utility of each criterion. In our study, NPV reflects the likelihood that patients who meet specific clinical criteria, such as pneumonia or COPD exacerbation, truly do not have PE, which is particularly relevant for assessing these criteria’s utility in ruling out PE in low-risk patients. Conversely, PPV represents the probability that patients who meet these criteria are diagnosed with PE, helping to evaluate each criterion’s predictive strength for a positive PE diagnosis within our cohort.

Our findings suggest that criteria with a high NPV, such as pneumonia, demonstrated significant diagnostic utility in low-risk patients. However, these results should be interpreted with caution, as a high NPV in low-risk populations does not necessarily apply to medium- or high-risk groups. Sensitivity analysis calculations were conducted rigorously to ensure that they accurately reflect each criterion’s diagnostic performance across different risk categories, and necessary adjustments were made to correct any discrepancies identified during the review process.

**Figure 2:**
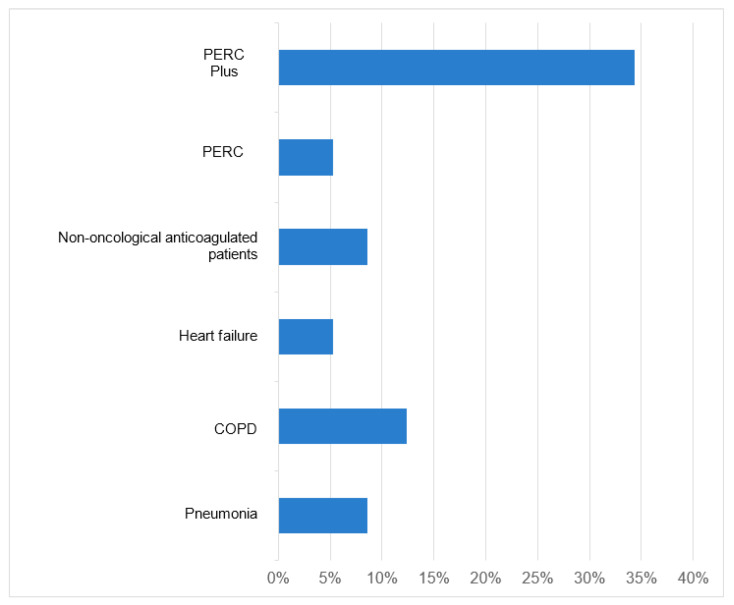
Percentage reduction of CT angiography with features with high negative predictive value and with PERC plus in patients with low Wells risk with a specificity of 100%.

The reduction in CT angiography use for conditions with high NPV was 9% for pneumonia, 12% for COPD, 5% for CHF, 9% for anticoagulated patients without oncological pathology, and 5% overall (Figure 2).

## 4. Discussion

Our findings highlight the importance of a structured approach to diagnosing pulmonary embolism in low-risk patients, particularly in emergency settings. The combination of the Wells score with additional clinical factors, such as the presence of objective alternative diagnoses, allowed for a reduction in unnecessary computed tomography angiographies in low-risk patients without compromising diagnostic accuracy. This is consistent with previous studies showing that the Wells score is a valuable tool in PE risk stratification [7,27]. However, our proposal to combine the Wells score with additional clinical factors, as performed in recent studies [33], further supports the potential to reduce unnecessary diagnostic tests, optimizing resources in emergency departments.

The presence of alternative diagnoses, such as pneumonia or COPD exacerbation, can reduce the likelihood of diagnosing PE, as suggested in earlier studies [42]. Additionally, the combination of the Wells scale and PERC has been shown to be particularly effective in elderly populations for ruling out PE, as demonstrated by Guo et al. [43]. The application of clinical decision rules like the Wells score and PERC in emergency settings has proven to significantly reduce unnecessary testing, as reported by Aydoğdu et al. [44,45]. The Wells score has also been shown to perform well in hospitalized patients with suspected PE, as evidenced by studies like Posadas-Martínez et al. [46].

In relation to COPD exacerbation, it is important to note that although PE can co-occur with COPD exacerbations, it should not be the primary diagnostic focus in patients who respond adequately to treatment for the exacerbation [47,48]. Our findings align with other studies suggesting that while PE prevalence can be significant in patients with COPD exacerbations, it does not justify imaging for PE in every case [26,49].

An important aspect not discussed previously is the need to validate risk scores in real-world scenarios. Many risk scores, including the Wells score, were derived in emergency department (ED) settings. Their application during hospitalization may require further validation and potential adjustments to account for different clinical environments and patient profiles. A recent study on syncope risk scores highlighted this issue, showing reduced validity for decisions during hospitalization and a need for adjustments with clinical variables [50]. This example emphasizes the importance of validating PE risk scores for inpatient use, as scores developed in the ED may not translate seamlessly to hospitalized populations without modifications.

Our results show that the Wells score performs well in identifying low-risk patients, with only 6 out of 210 patients in this category diagnosed with PE. This supports the use of the Wells score for risk stratification in the emergency setting. However, this study is limited to emergency department patients and does not include performance analysis in hospitalized patients, as these data are beyond our study’s scope. The good performance of the Wells score in the low-risk group in our study could be attributed to several factors. First, careful patient selection in the emergency department, where the score is applied only when there are clear indications of PE, may reduce the incidence of positive diagnoses in low-risk groups. Additionally, the absence of severe concurrent diagnoses in these patients could influence the accuracy of the Wells score in this specific setting, as comorbidities and aggravating conditions are less common in the emergency department than in hospitalized patients. It is also possible that there is a preventive bias in the emergency department, where physicians may be more inclined to rule out PE in patients with atypical symptoms or clear alternative diagnoses.

The role of D-dimer testing in low-risk patients is an important consideration when aiming to reduce unnecessary imaging in suspected PE cases. In patients classified as low-risk according to the Wells score, a negative D-dimer result has been shown to reliably exclude PE, potentially preventing the need for further diagnostic testing such as CT angiography. This approach aligns with recent guidelines and studies that advocate for the use of D-dimer testing in combination with clinical decision rules to enhance diagnostic accuracy and reduce resource use in emergency settings [20,28]. Although D-dimer was not included as a primary factor in our study, future research could investigate its combined use with clinical criteria and objective alternative diagnoses (such as pneumonia or COPD exacerbations) to further streamline the diagnostic process for low-risk patients.

It is notable that the combination of the PERC with additional clinical factors, referred to as PERC plus, showed 100% sensitivity and negative predictive value in low-risk patients according to the Wells score. This is consistent with previous studies on the utility of PE rule-out criteria, highlighting the safety of using clinical criteria to reduce CT utilization in low-risk patients [33,44].

Nevertheless, one of the limitations of our study is its retrospective, single-center design, which may introduce selection bias and limit the generalizability of the results. While clinical factors with high NPV appear promising, further validation of this combination in prospective studies with larger cohorts is necessary to confirm its broader applicability.

The appropriate use of diagnostic resources in the management of PE remains a challenge, and our study contributes to this body of knowledge by demonstrating that the combination of clinical tools and objective factors can reduce reliance on costly imaging without compromising patient safety [28,32].

## 5. Conclusions

The PERC plus scale shows potential in excluding PE in patients classified as low risk by the Wells scale, potentially reducing the need for further tests in both outpatient and inpatient settings. Combining objective clinical criteria with the PERC scale could improve diagnostic accuracy and reduce unnecessary testing, thus enhancing patient management and resource utilization in acute care environments.

## Figures and Tables

**Table 1:** Modified Wells Scale for Pulmonary Thromboembolism.

Characteristics	Value
Clinical symptoms of deep vein thrombosis	3
Other diagnoses less likely than PE	3
Heart rate greater than 100 bpm	1.5
Immobilization or surgery within the last 4 weeks	1.5
Deep vein thrombosis or previous pulmonary thromboembolism	1.5
Hemoptysis	1
Cancer	1

PE: Pulmonary thromboembolism; bpm: Beats per minute.

**Table 2:** PERC.

Characteristics
- Under 50 years of age
- Pulse < 100 lpm (in tachycardia)
- Absence of hypoxia (SatO_2_ > 95%)
- No history of pulmonary embolism or deep vein thrombosis
- No trauma or recent surgery
- Absence of hemoptysis
- Does not use estrogen therapy
- Absence of swelling in the legs

PERC: Pulmonary Embolism Rule-Out Criteria; SatO_2_: Oxygen; saturation; bpm: Beats per minute.

**Table 3:** Definition of objective variables with high negative predictive value.

Pneumonia	Presence of Fever. Radiological Pattern Compatible with Respiratory Infection and Dyspnea or Cough
Exacerbation/suspicion of COPD	History of smoking, dyspnea, and cough
Heart failure	Presence of radiological pattern compatible with heart failure and dyspnea or orthopnea
Anticoagulation in non-cancer patients	Previous anticoagulation in non-cancer patients
PERC	Those patients who meet the PERC mentioned in Table 2

COPD: Chronic obstructive pulmonary disease; PERC: Pulmonary; Embolism Rule-Out Criteria.

**Table 4:** Characteristics of the population.

Variable	Total	Risk According to the Wells Scale			*p*
	*n* = 399	Low	Intermediate *n* = 150	High	
		*n* = 210		*n* = 39	
Age, years (SD)	65 ± 16	65 ± 16	65 ± 16	64 ± 16	0.731
Women, %	214 (53.60%)	118 (56.20%)	79 (52.70%)	17 (43.60%)	0.334
Comorbidities					
Patients without comorbidities, %	46 (11.50%)	27 (12.90%)	14 (9.30%)	5 (12.80%)	0.567
Smoking, %	137 (34.30%)	83 (39.50%)	49 (32.70%)	5 (12.80%)	0.005
Ischemic heart disease, %	55 (13.79%)	34 (16.20%)	17 (11.30%)	4 (10.30%)	0.335
COPD, %	48 (12.00%)	34 (16.20%)	14 (9.30%)	0 (0.00%)	0.007
Renal insufficiency, %	17 (4.30%)	9 (4.30%)	8 (5.30%)	0 (0.00%)	0.34
Oncological pathology, %	121 (30.30%)	48 (22.90%)	56 (37.30%)	17 (43.60%)	0.002
Summa of comorbidities, SD	0.885 ± 0.32	0.871 ± 0.34	0.907 ± 0.29	0.872 ± 0.34	0.569
Symptoms					
Dyspnea, %	269 (67.40%)	133 (63.30%)	108 (72.00%)	28 (71.80%)	0.186
Hemoptysis, %	11 (2.80%)	5 (2.40%)	5 (3.30%)	1 (2.60%)	0.86
Pleuritic pain, %	154 (38.60%)	84 (40.00%)	57 (38.00%)	13 (33.30%)	0.721
Lower limb pain, %	43 (10.80%)	5 (2.40%)	18 (12.00%)	20 (51.30%)	<0.001
Cough, %	115 (28.80%)	76 (36.20%)	33 (22.00%)	6 (15.40%)	0.002
Faint, %	15 (3.80%)	5 (2.40%)	8 (5.30%)	2 (5.10%)	0.316
Fever, %	66 (16.50%)	39 (18.60%)	23 (15.30%)	4 (10.30%)	0.386
Orthopnea, %	25 (6.30%)	12 (5.70%)	11 (7.30%)	2 (5.10%)	0.784
Vital signs					
Tachycardia > 100 lpm, %	105 (26.30%)	36 (17.10%)	55 (36.70%)	14 (35.90%)	<0.001
SBP < 90 mmHg, %	21 (5.30%)	9 (4.30%)	8 (5.30%)	4 (10.30%)	0.308
Saturation < 94%, %	185 (46.40%)	95 (45.20%)	71 (47.30%)	19 (48.70%)	0.882
Variables with high a priori negative predictive value for PE					
PERC, %	13 (3.30%)	11 (5.20%)	2 (1.30%)	0 (0.00%)	0.058
Pneumonia, %	27 (6.80%)	18 (8.60%)	9 (6.00%)	0 (0.00%)	0.132
COPD flare-up, %	40 (10.00%)	26 (12.40%)	13 (8.70%)	1 (2.60%)	0.135
Heart failure, %	18 (4.50%)	11 (5.20%)	5 (3.30%)	2 (5.10%)	0.679
Non-oncology patients on anticoagulation, %	29 (7.30%)	18 (8.60%)	11 (7.30%)	0 (0.00%)	0.166
CT angiography result					
Pulmonary thromboembolism, %	139 (34.80%)	6 (2.90%)	95 (63.30%)	38 (97.40%)	<0.001
Patients with CT angiography performed in the emergency department, %	324 (81.20%)	166 (79.00%)	121 (80.70%)	37 (94.40%)	0.066

COPD: Chronic obstructive pulmonary disease; SBP: Systolic blood pressure; bpm: Beats per minute; PERC: Pulmonary Embolism Rule-Out Criteria; PE: Pulmonary embolism; CT angiography: Computed tomography angiography; SD: Standard deviation.

**Table 5:** Population characteristics as a function of thoracic CT angiography.

Variable	PE	Not PE	*p*
Age, years (SD)	66 ± 17	64 ± 16	0.821
Women, %	65 (46.80%)	149 (57.30%)	0.44
Positive CT angiography results in the emergency department, %	124 (89.20%)	200 (76.9%)	0.003
Comorbidities			
Patients without comorbidities, %	16 (11.50%)	30 (11.50%)	0.993
Smoking, %	31 (22.30%)	106 (40.80%)	<0.001
Ischemic heart disease, %	16 (11.50%)	39 (15.00%)	0.335
COPD, %	9 (6.50%)	39 (15.00%)	0.013
Renal insufficiency, %	3 (2.20%)	14 (5.40%)	0.128
Oncological Pathology, %	45 (32.40%)	76 (29.20%)	0.515
Summary of comorbidities (SD)	0.885 ± 0.320	0.885 ± 0.320	0.993
Symptoms			
Dyspnea, %	101 (72.70%)	168 (64.60%)	0.102
Hemoptysis, %	4 (2.90%)	7 (2.70%)	0.914
Pleuritic pain, %	60 (43.20%)	94 (36.20%)	0.17
Lower limb pain, %	31 (22.30%)	12 (4.60%)	<0.001
Cough, %	21 (15.10%)	94 (36.20%)	<0.001
Faint, %	6 (4.30%)	9 (3.50%)	0.674
Fever, %	17 (12.20%)	49 (18.80%)	0.09
Orthopnea, %	6 (4.30%)	19 (7.30%)	0.24
Vital signs			
Tachycardia > 100 lpm, %	46 (33.10%)	59 (22.70%)	0.025
SBP < 90 mmHg, %	8 (5.80%)	13 (5.00%)	0.747
SatO_2_ < 94%, %	79 (56.80%)	106 (40.80%)	0.002
Wells scale			
Low risk, %	6 (4.30%)	204 (78.50%)	<0.001
Moderate risk, %	95 (68.30%)	55 (21.20%)	
High risk, %	38 (27.30%)	1 (0.40%)	
Variables with high a priori negative predictive value for PE			
PERC, %	0 (0.00%)	13 (5.00%)	0.007
Pneumonia, %	5 (3.60%)	22 (8.50%)	0.065
COPD flare-up, %	6 (4.30%)	34 (13.00%)	0.006
Heart failure, %	7 (5.00%)	11 (4.20%)	0.712
Non-oncology patients on anticoagulation, %	5 (3.60%)	24 (9.20%)	0.039

COPD: Chronic obstructive pulmonary disease; SBP: Systolic blood pressure; SatO_2_: Oxygen saturation; bpm: Beats per minute; PERC: Pulmonary Embolism Rule-Out Criteria; PE: Pulmonary embolism; CT angiography: Computed tomography angiography; SD: Standard deviation.

**Table 6:** Study of the ability to discriminate factors with high a priori negative predictive value for the diagnosis of pulmonary thromboembolism.

	Risk	Sensitivity	Specificity	PPV	NPV	% CT Angiography Reduction
Pneumonia	Low	100.00%	8.82%	3.13%	100.00%	8.57%
	Middle	94.74%	7.27%	63.83%	44.44%	2.67%
	High	100.00%	0.00%	97.44%	0.00%	0.00%
COPD	Low	100.00%	12.75%%	3.26%	100.00%	12.38%
	Middle	94.74%	14.55%	65.69%	61.54%	5.33%
	High	97.37%	0.00%	97.37%	0.00%	0.00%
Heart failure	Low	100.00%	5.40%	3.02%	100.00%	5.24%
	Middle	94.74%	0.00%	62.07%	0.00%	0.00%
	High	94.74%	0.00%	97.30%	0.00%	0.00%
Non-oncological anticoagulated patients	Low	100.00%	8.82%	3.13%	100.00%	8.57%
	Middle	94.74%	10.90%	64.75%	54.55%	4.00%
	High	100.00%	0.00%	97.44%	0.00%	0.00%
PERC	Low	100.00%	5.39%	3.02%	100.00%	5.24%
	Middle	100.00%	3.64%	64.19%	100.00%	1.33%
	High	100.00%	0.00%	97.44%	0.00%	0.00%
PERC plus	Low	100.00%	34.31%	4.29%	100.00%	34.30%
	Middle	83.16%	25.45%	65.83%	46.67%	25.50%
	High	92.11%	0.00%	97.22%	0.00%	0.00%

COPD: Chronic obstructive pulmonary disease; PERC: Pulmonary Embolism Rule-Out Criteria; PPV: Positive predictive value; NPV: Negative predictive value; CT angiography: Computed tomography angiography; “Positive CT angiography results in the emergency department, %” indicates the percentage of patients who had a positive result for pulmonary embolism on CT angiography performed in the emergency department, distinguishing between those diagnosed with PE and those without PE based on other clinical factors.

## Data Availability

The data will be accessible upon direct request to the authors.
